# Real-time observation of topological defect dynamics mediating two-dimensional skyrmion lattice melting

**DOI:** 10.1038/s41565-025-01977-2

**Published:** 2025-08-04

**Authors:** Raphael Gruber, Jan Rothörl, Simon M. Fröhlich, Maarten A. Brems, Fabian Kammerbauer, Maria-Andromachi Syskaki, Elizabeth M. Jefremovas, Sachin Krishnia, Asle Sudbø, Peter Virnau, Mathias Kläui

**Affiliations:** 1 Institute of Physics, Johannes Gutenberg-Universität Mainz, Mainz, Germany; 2 Singulus Technologies AG, Kahl am Main, Germany; 3 Center for Quantum Spintronics, Department of Physics, Norwegian University of Science and Technology, Trondheim, Norway

**Keywords:** Phase transitions and critical phenomena, Spintronics

## Abstract

Topological defects are the key feature mediating two-dimensional phase transitions. However, both resolution and tunability have been lacking to access the dynamics of these transitions in the various two-dimensional systems explored. Skyrmions in magnetic thin films are two-dimensional, topologically non-trivial quasi-particles that provide rich dynamics as well as tunability as an essential ingredient for the control of their phase behaviour. With dynamic Kerr microscopy, we directly capture the melting of a confined two-dimensional magnetic skyrmion lattice in a Ta/CoFeB/Ta/MgO/Ta magnetic multilayer system with high resolution in real time and real space. By the applied magnetic field, we tune the skyrmion size and effective temperature on the fly to drive the two-step melting through an intermediate hexatic regime between the solid lattice and the isotropic liquid. We quantify the characteristic occurrence of topological defects mediating the transitions and reveal the dynamics of the lattice dislocations. The full real-time and real-space imaging reveals the diffusion coefficient of dislocations, which is two orders of magnitude higher than that of skyrmions.

## Abstract (web summary)

Dynamic Kerr microscopy enables the tracking of the two-step melting of a magnetic skyrmion lattice from a two-dimensional solid through an intermediate hexatic regime to an isotropic liquid and provides direct insights in the occurrence and dynamics of lattice dislocations, the defects that mediate melting.

## Main

Magnetic skyrmions are topologically non-trivial chiral spin structures exhibiting quasi-particle behaviour^1–3^. Besides being ideal candidates for low-power applications in data storage and processing^4–13^, skyrmions hosted in nanometre-thin films^3^ are a flexible model system for studying two-dimensional (2D) system properties^14–16^, particularly 2D phase transitions.

The Kosterlitz–Thouless–Halperin–Nelson–Young (KTHNY) theory^17–21^ describes 2D melting from a solid with translational quasi-long-range order (QLRO) to a disordered, isotropic liquid in two steps via an intermediate hexatic phase with orientational QLRO only. The two KTHNY phase transitions are associated with the unbinding and proliferation of pairs of topological defects of the lattice and have been observed in several systems and experiments, including colloids^22,23^, superconducting vortices^24,25^ and skyrmions^14,26,27^. However, in all these previous experimental investigations, resolution^14,15,24,25^ or tunability^15,22,23,27^ of the system have been insufficient to drive the system through phase transitions and elucidate the dynamics of melting, including the defect evolution in real time and real space.

Skyrmions in Ta/CoFeB/Ta/MgO magnetic thin films form 2D lattices with their purely repulsive interaction potentials^28–30^, exhibiting rich Brownian dynamics^7,31,32^ and providing the required tunability of size and diffusivity^7,32–34^ to control transitions on the fly. Additionally, in Kerr microscopy, their dynamics is directly accessible in real time and real space, which is challenging in many other techniques^14,15^. However, pinning effects in the form of a non-flat-energy landscape in the magnetic multilayers have hampered the formation of QLRO of skyrmion lattices^15,16,26,35,36^. Therefore, for sufficiently low pinning, the structural disorder may be small enough to allow for translational order and enhancing the phase space of the hexatic phase^26,35^, enabling better feasibility to study the phases and transitions.

In this study, we exploit the on-the-fly tunability of our 2D skyrmion lattice to melt the system to disorder in a two-step process using two independent methods: (1) by shrinking the skyrmions and reducing the packing fraction and (2) by increasing the skyrmions’ diffusivity corresponding to an effective temperature. We stabilize the skyrmion lattice in a low-pinning Ta/CoFeB/Ta/MgO magnetic thin-film stack^7,33,34^ at 333.5 K using hexagonal geometric confinement providing commensurate boundary conditions^37^ for the lattice formation, and we identify the two-step melting by spatial and time correlation functions. With high-resolution Kerr microscopy in real time and real space, we capture the topological defect dynamics and find that the diffusion coefficient of dislocations is two orders of magnitude larger than that of skyrmions. Therefore, the rich dynamics of the system yields powerful insights into the formation and dissociation of topological defects, which is the key feature mediating the melting transitions in two dimensions—but has so far been experimentally inaccessible.

## Two-step skyrmion lattice melting

**Fig. 1:**
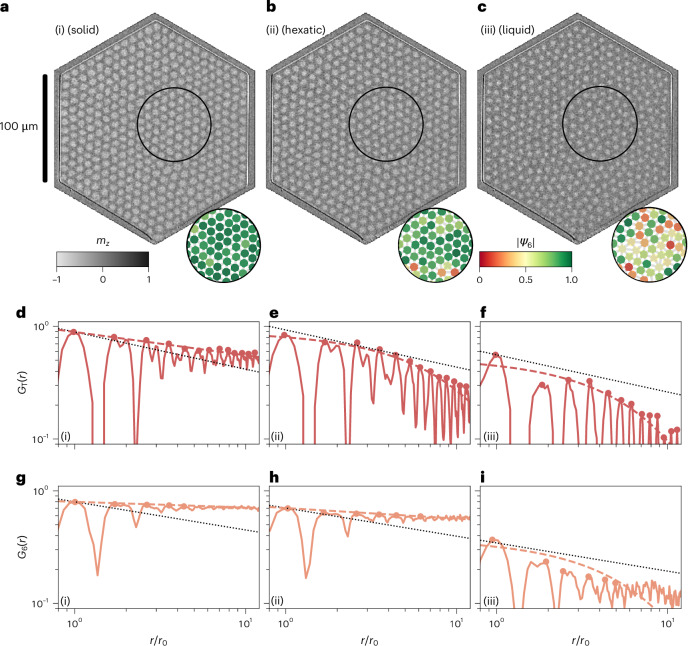
Skyrmion lattices with different order. **a**–**c**, Kerr microscopy images of 401 skyrmions comprising lattices in hexagonal geometric confinement: snapshots (i)–(iii) at 60 µT (**a**), 84 µT (**b**) and 114 µT (**c**); greyscale contrast represents the OOP magnetization *m*_*z*_. The insets visualize the local order parameter |*ψ*_6_| per skyrmion in the black circle. **d**–**f**, Translational correlation functions *G*_T_(*r*) as a function of distance *r* in units of nearest-neighbour distances *r*_0_. The dashed line represents the power-law (**d**) and exponential (**e** and **f**) envelope fits for snapshots (i)–(iii), and the dots are fitted points. The dotted black line is the power law with the critical exponent *η*_T_ = 1/3 for reference. Although the data in **d** decay algebraically with an exponent smaller than 1/3, the data in **e** and **f** decay exponentially. **g**–**i**, Analogous orientational correlation functions *G*_6_(*r*) as a function of distance *r*/*r*_0_ (solid orange line), envelope fit (dashed line) and fitted points (dotted line) for snapshots (i)–(iii). The dotted black line represents the power law with critical exponent *η*_6_ = 1/4. Although the data in **g** and **h** exhibit power-law behaviour with *η*_6_ smaller than 1/4, the data in **i** yields a faster decay. Source data

We stabilize a skyrmion lattice in a hexagonal geometric confinement of 100-µm edge length in a Ta(5 nm)/Co_20_Fe_60_B_20_(0.9 nm)/Ta(0.07 nm)/MgO(2 nm)/Ta(5 nm) stack. We observe the skyrmions in real time and real space with Kerr microscopy^7^ (Supplementary Video 1)^38^. In Fig. 1a–c, we show Kerr microscopy snapshots (i)–(iii) at different magnetic out-of-plane (OOP) field, which controls the skyrmion size^33,39,40^ and, thus, the packing fraction.

We determine the local order parameter *ψ*_6_ (Fig. 1a–c, insets) and calculate the translational (Fig. 1d–f) and orientational (Fig. 1g–i) correlation functions to analyse the ordering. We fit the correlation functions *G*_T_(*r*) and *G*_6_(*r*) with a power-law decay, yielding exponents *η*_T_ and *η*_6_, respectively. Furthermore, we perform an exponential fit to determine the corresponding correlation lengths *ξ*_T_ and *ξ*_6_ (Methods). Despite our finite system, we use the terms solid, hexatic and liquid measured in analogy to the KTHNY theory to distinguish between the different regimes.

For the densely packed skyrmions in snapshot (i), the translational correlation *G*_T_(*r*) decays algebraically with an exponent *η*_T_ smaller than 1/3. The orientational correlation *G*_6_(*r*) is almost constant. Hence, the system exhibits translational and orientational orders, which we identify as solid. For snapshot (ii), *G*_T_(*r*) decays exponentially, whereas *G*_6_(*r*) is still decaying algebraically with an exponent *η*_6_ smaller than 1/4. Thus, the translational order has vanished, whereas the orientational order persists; we identify this as hexatic. In snapshot (iii), both *G*_T_(*r*) and *G*_6_(*r*) decay faster with the critical exponent; therefore, we classify this unordered state as liquid.

Finite-size effects occurring for the correlation functions, such as deviations for larger distances and the unclear exponential behaviour for snapshot (iii) (Fig. 1i) are described in Supplementary Note 1 and Supplementary Fig. 1. Furthermore, in the Thiele model simulations, we see that hexagonal confinements indeed stabilize ordering, particularly for smaller systems and with commensurability (Supplementary Note 2 with Supplementary Fig. 2). Regarding the qualitative processes and critical exponents, however, neither simulations nor experiments appear to be affected noticeably by finite-size effects. Therefore, we use the confinement for lattice stabilization and preventing local pinning centres from macroscopically hampering the lattice order^15,16,26^. Therefore, we reveal intrinsic effects and dynamics during a two-step melting expected from the KTHNY theory.

In contrast to other systems in which 2D phases have been observed^14,15,22^, our system is highly flexible in the sense that we can tune the skyrmions’ size^31,33^ and mobility^34^ on the fly by the OOP magnetic field, in addition to the thermal Brownian-like diffusion^7^. Together with direct real-time observation in Kerr microscopy, this allows for a full real-time and real-space analysis of the dynamics associated with 2D phase transitions and critical phenomena, which have not been revealed in other systems. In the following, we perform the time-resolved quantitative analysis of the observed melting.

## Real-time quantification of the melting

**Fig. 2:**
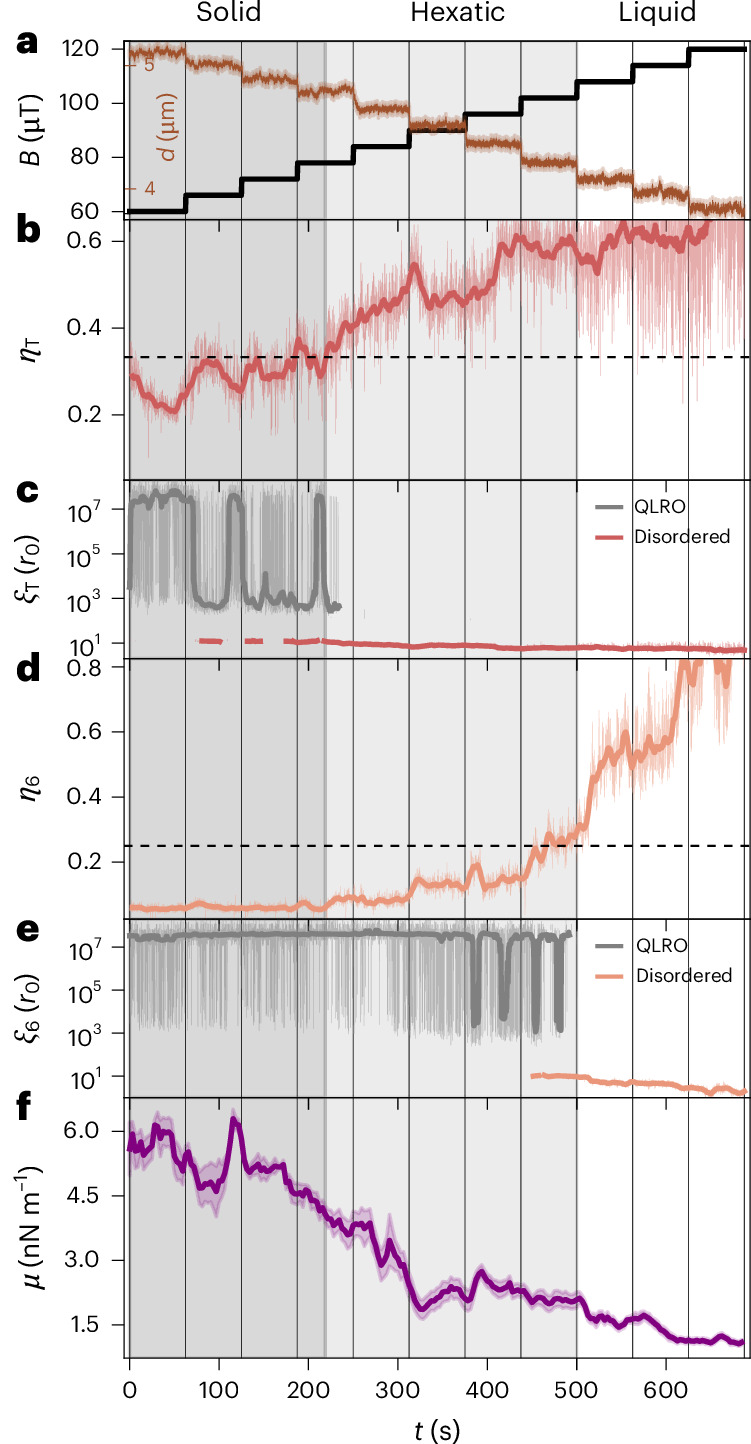
Time-resolved quantification of the melting. **a**, The applied magnetic field *B* (black) is increased stepwise, directly causing the skyrmion diameter *d* (brown, average and standard deviation) to decrease. The vertical black lines delimit the intervals of constant OOP fields in all panels. **b**, Exponent *η*_T_ of *G*_T_ and rolling mean over 6.25 s. The dashed horizontal line marks the critical exponent of 1/3. **c**, Correlation length *ξ*_T_ of *G*_T_ and rolling median over 6.25 s. *ξ*_T_ is well beyond the system size in the critical (QLRO) regime (grey) and within the system size in the disordered regime (red). **d**, Exponent *η*_6_ of *G*_6_ and rolling mean over 6.25 s. The dashed horizontal line marks the critical exponent of 1/4. **e**, Correlation length *ξ*_6_ of *G*_6_ and rolling median over 6.25 s for the QLRO (grey) and disordered (orange) regimes. The dark-grey, light-grey and white backgrounds represent the solid, hexatic and liquid regimes, respectively. **f**, Shear modulus *μ* of the skyrmion lattice determined from fitting the local lattice deformations on a logarithmic scale. Fit (solid line) and standard deviation of the fit (shaded area) are shown for sliding window averages over 6.25 s (100 frames). Source data

To capture the full melting process, we stepwise increase the magnetic field to shrink the skyrmions and, thus, reduce their packing fraction. We observe the evolution of lattice order—disorder emerging from the field steps and fluctuations in the intervals of constant field—to fully capture the melting dynamics in a quasi-equilibrium process (Methods). In Fig. 2a, we visualize the OOP field (black) and the corresponding skyrmion diameter (brown). The black vertical lines represent the times at which the field changes in all panels. Since the shrinking skyrmions have effectively more space available, the number of accessible microstates and, thus, the configurational entropy increase, which we exploit to drive the system to disorder.

Figure 2b shows the time evolution of *η*_T_; the black dotted line marks its critical value of 1/3. We find that initially, the exponent is below the critical value, signalling translational QLRO, which we identify as the solid regime. After 70 s, the exponent starts fluctuating around the critical value. At around 225 s, after further shrinking steps, *η*_T_ permanently exceeds 1/3. However, the power-law exponent is only well defined in the critical, ordered phase delimited by the critical exponent (*η*_T_ = 1/3), where the decay becomes solely exponential. Figure 2c shows the corresponding correlation length *ξ*_T_. Note that *ξ*_T_ must be determined differently for the critical (QLRO) and disordered regimes (Fig. 2c). In the solid regime, we accordingly observe *ξ*_T_ being orders of magnitude beyond the system size—effectively corresponding to divergence as predicted by the KTHNY theory^18,19^, highlighting the quality of the power-law fit. Reaching the critical exponent, the correlation length drops below the system size. Hence, the solid character given by translational QLRO has vanished.

Analogously, we plot *η*_6_ and the associated correlation length *ξ*_6_ (Fig. 2d,e, respectively). We see that *η*_6_ starts well below its critical value of 1/4 (black dots) in the solid, meaning *G*_6_ is almost constant (in contrast to *G*_T_). At the point where the translational order vanishes, however, the orientational order persists as *η*_6_ still stays below its critical value of 1/4, which we identify as hexatic. In the hexatic regime, the exponent *η*_6_ grows significantly until reaching 1/4 eventually, denoting the transition to a liquid. It fluctuates around the critical value after around 450 s and permanently exceeds it after 490 s. We visualize the three determined regimes by the grey shading in Fig. 2.

**Extended Data Fig. 1:**
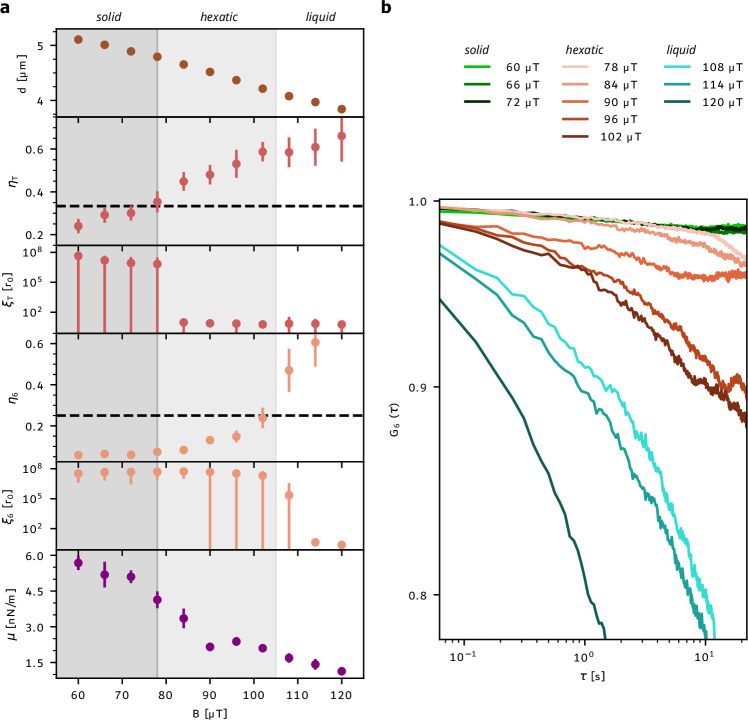
Quantification of Melting – Dependence on Magnetic Field. **a** Increasing the magnetic field *B* causes the skyrmion diameter *d* (mean and negligible standard error of the mean) to shrink. Correspondingly, we plot the time-average over the fluctuations (error bar: standard deviation) of the ordering quantities presented in Fig. 2 for the 62.5 s interval (1000 frames) recorded at every field value: exponent *η*_T_ of and correlation length *ξ*_T_ of the translational correlation function *G*_T_(*r*), exponent *η*_6_ of and correlation length *ξ*_6_ of the orientational correlation function *G*_6_(*r*), and shear modulus *μ*. **b** Orientational time correlation function *G*_6_(*τ*) for every time delay *τ* (sliding window average) at every field value. We see a clear separation between the correlation in the three different regimes. While *G*_6_(*t*) is almost constant in the solid regime, it decays notably in the hexatic regime and much faster in the liquid, in good agreement with theory. The predicted critical value of *η*_*τ*_=1/8 for an infinite system is however too large to match our scenario. We can attribute the enhanced time correlation in our experiment to effects of the confinement and the non-flat energy landscape. Source data

Since we drive the melting by shrinking the skyrmions with the applied magnetic field, we additionally present the respective data based on the applied magnetic field (Extended Data Fig. 1)—as both time average and time-dependent orientational correlation *G*_6_(*τ*) for every field interval, showing good agreement with theory^23^.

**Extended Data Fig. 2:**
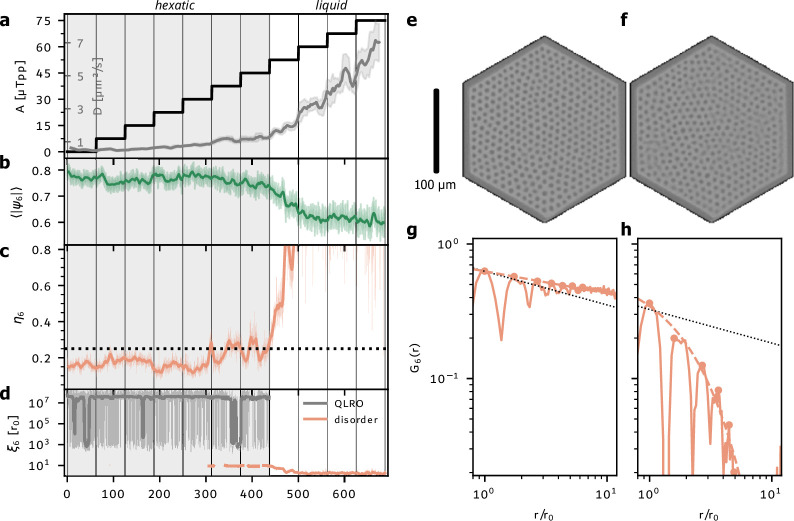
Lattice Melting by Increasing Skyrmion Diffusivity. **a** The amplitude *A* (peak-to-peak at 100 Hz) of the oscillating applied field is increased in steps (black) while the offset field is kept constant at 102 µT. This causes the diffusion coefficient *D* of the 378 skyrmions to increase (gray, rolling mean and standard deviation). With increasing diffusivity, skyrmions start annihilating. Black vertical lines denote the field steps in all sublots. **b** Average absolute value 〈|*ψ*_6_|〉 of the local order parameter, for each frame (shaded) and rolling average over 6.25 s (solid line). **c** Exponent *η*_*6*_ of *G*_6_ and rolling mean over 6.25 s. The dotted horizontal line marks the critical exponent of 1/4. We find that tuning the diffusivity, we can drive the *η*_*6*_ from well below to above the critical value of 1/4. **d** Correlation length *ξ*_6_ of *G*_6_ and rolling median over 6.25 s (100 frames). **e**, **f** Kerr images averaged over 5 s at (**e**) *t* = 75 s and (**f**) *t* = 575 s. The smeared-out contrast visualizes the increased diffusivity in (**f**). **g**, **h** Orientational correlation functions *G*_6_ for (**e**) and (**f**), respectively. The black dots represent a power-law decay with the critical exponent of 1/4. The dashed line is the envelope fit and the dots are the points used for the fit. While in the hexatic case in (**g**), *G*_6_ decays algebraically, it is now exponential in the liquid case shown in (**h**). Source data

To further underline the robustness of our analysis, we go beyond the entropy-mediated melting due to skyrmion shrinking and exploit the tunability of our system further to provide a second, independent approach to drive the system across disordering transitions. By magnetic OOP-field oscillations, we increase the diffusivity^34^ (that is, the effective temperature of the system; Supplementary Video 2)^38^ and, therefore, induce melting (Extended Data Fig. 2). The results are consistent for the two approaches; however, in the case of field oscillations present, we expect the emergence of non-equilibrium properties due to the permanent driving of the skyrmion size.

**Extended Data Fig. 3:**
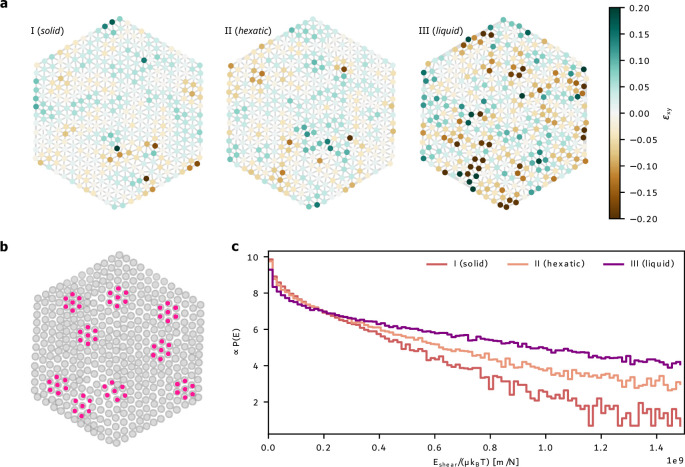
Shear Analysis of the Lattice. **a** Shear components *ε*_xy_ of the determined local lattice deformations for snapshots (i)-(iii) from Fig. 1a-c. During the melting, larger components of the strain tensor become possible and more likely. **b** Visualization of determined local lattice deformation at *t* = 0. The gray circles denote the observed skyrmion positions. The pink dots represent the locally deformed lattice at exemplary sites: For every skyrmion position, a deformation of a local hexagonal lattice (up to the first nearest neighbors) is fitted to match the neighboring positions best. *ε*_xy_ is extracted as the asymmetric component of the deformation matrix. **c** Every shear component over the area spanned by the nearest neighbors is associated with a shear energy *E*_shear_ in units of the shear modulus *μ* and *k*_B_*T*, which is related to the Boltzmann energy distribution *P*(*E*). The slope of the distribution determines the shear modulus *μ*. The shear energy contributions are shown for the snapshots (i)-(iii) using statistics of 12.5 s. Source data

Finally, we can even probe the shear modulus *μ* (refs. ^41,42^) by analysing local lattice deformations as the characteristic behaviour associated with melting transitions is also exhibited by elastic constants. In Fig. 2f, we show that *μ* is approximately constant in the solid. When translational QLRO vanishes, *μ* decreases significantly. However, *μ* is not vanishing since we must assume linear elasticity in our analysis, which becomes less applicable when reducing the packing fraction. Yet, qualitatively, the resulting shear modulus clearly supports our conclusions. In Extended Data Fig. 3, we present example shear components and their energies.

Hence, we demonstrate that we can drive a finite lattice from solid order to a liquid via the hexatic regime, particularly image the melting process directly with high time resolution. This allows us to access the dynamics of every skyrmion quasi-particle. With the full information of the individual skyrmion trajectories, we can uniquely identify the topological lattice defects and probe their dynamics, which is typically not accessible. The topological defects are the key feature of 2D melting, as described in the KTHNY theory. Therefore, we need to quantify the occurrence and dynamics of those lattice defects to fully understand the transitions.

## Topological defects dynamics

The formation and dissociation of topological defects is the key feature of 2D melting in the KTHNY theory. Our system allows us to observe the defect dynamics, gaining insights into the melting process in a unique way. The thermally activated diffusion of skyrmions induces rich dynamics including lattice fluctuations and defect formations on sub-second timescales and dynamic imaging allows us to unambiguously identify the topological defects (Supplementary Video 3). Due to the time resolution of 62.5 ms, we can further analyse the dynamics of the topological defects. In particular, we can tune the dynamics of both skyrmions and defects by the applied magnetic field. We note that previous observations of 2D phase transitions lack sufficient resolution^14,24^ or tunability of order and its fluctuations^15,22,23,27^ to explicitly investigate the defect dynamics.

**Fig. 3:**
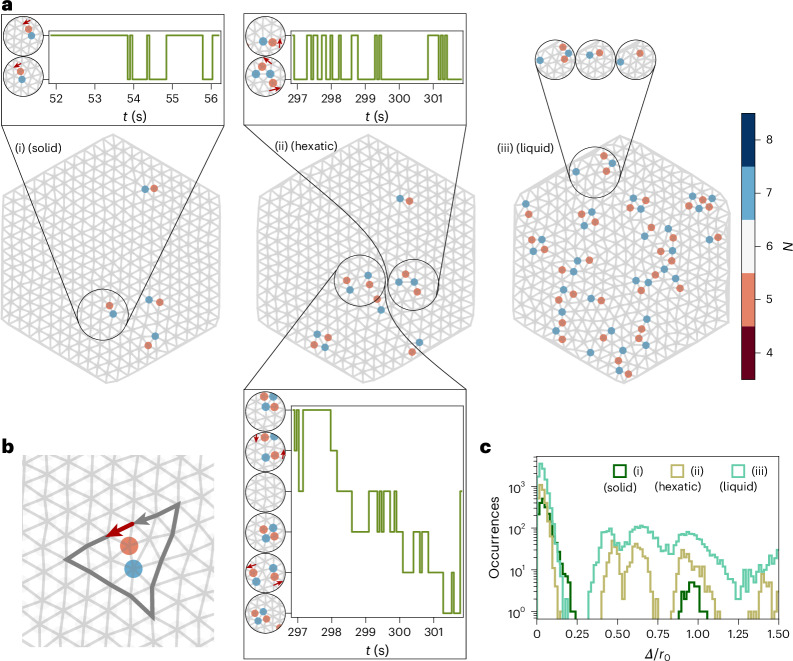
Fluctuations of topological defects. **a**, Lattice defects of snapshots (i)–(iii), coloured by the number of lattice neighbours *N*. Snapshots (i)–(iii) from Fig. 1a–c are used, which correspond to the images of melting shown in Fig. 2 at *t*_(i)_ = 50.0 s, *t*_(ii)_ = 301.6 s and *t*_(iii)_ = 576.3 s, respectively. The grey net shows the nearest-neighbour connections between the experimental skyrmions. The insets show how defect configurations can change between discrete states, and the attached green step plots visualize the fluctuations between those states in measurement time *t*. Example Burgers vectors are drawn as red arrows. **b**, Four lattice vectors (grey) along each crystal direction would yield a closed path in a perfect lattice. However, to surround a dislocation counterclockwise, an additional lattice vector (red) is missing; it is identified as the Burgers vector. **c**, Histogram of dislocation displacements *Δ* within 62.5 ms (one frame) compared for snapshots (i)–(iii) (using the statistics of 31 s). Source data

In Fig. 3a, we present the topological defect map for the previously defined snapshots (i)–(iii). In the dense-packed (solid) snapshot (i), only four dislocations exist. We identify those dislocations as a consequence of the incommensurate number of skyrmions (Supplementary Note 3 and Supplementary Fig. 3), combined with the underlying energy landscape of the sample^32,33^. These dislocations remain stable and may only hop between the nearest-neighbour sites. An example of such a fluctuating dislocation is shown in the clipped inset connected to the black circle (Fig. 3a). The dislocation fluctuates back and forth between two discrete states. The discrete states are represented on the vertical axis in the black boxes in Fig. 3a, where the green line shows the temporal fluctuation between the states in measurement time *t*. We see that the Burgers vector (red arrows in the insets) associated with the dislocation is preserved when the dislocation hops. An example construction of the Burgers vector is shown in Fig. 3b. In addition to the four dislocations present in the solid, dislocation pairs can form spontaneously and annihilate again within a short time. The dislocation pairs are the characteristic clusters in the solid phase and have opposite Burgers vectors, which cancel out to zero.

In the 5-s time window around the hexatic snapshot (ii), new defects appear and exhibit more dynamics, also indicated in Fig. 3a. In the corresponding inset, the fluctuations between the identified states are showcased as a step function. The top inset shows how a dislocation can rearrange into two dislocations. Indeed, the total Burgers vector is conserved during the rearrangement. Furthermore, the second (bottom) inset for snapshot (ii) reveals how a dislocation pair can be created and annihilated, split up, merge and, therefore, move. These local fluctuations result in at least the six presented states. Note that during the fluctuation, the total Burgers vector is always zero. However, the opposing vectors can change direction as the dislocation pair can split into either two horizontally or two vertically oriented dislocations. By the controlled manipulation of the energy landscape^43,44^, such fluctuations could, in principle, become deterministic and, thus, transport information rapidly with very low power in specially designed devices. By tailoring the boundary conditions and comparing the skyrmion lattices in distorted hexagons, even topological defect dynamics under shear^45^ could be experimentally explored. The defect interaction potential, however, remains an open question as our limited system size does not provide sufficient statistics.

In the disordered liquid snapshot (iii), the created and constantly rearranging defects form a complex pattern consisting of several defect clusters. The clusters still span dislocations and square dislocation pairs, but also isolated defects, linear defect chains and more complex configurations. The corresponding inset in Fig. 3a shows an example of how a dislocation can split into two disclinations or rearrange into an isolated defect plus a chain of three defects as a key feature of the liquid regime. Since dislocations and clusters, in general, can rearrange, split and merge in many ways and, therefore, increasingly interact with neighbouring clusters, the number of observed states as well as their dynamics increases drastically. Eventually, the complexity does not allow us anymore to determine specific states and their fluctuations in time within a clipped region only.

This becomes more apparent when we investigate the dynamics of defect pairs. To this purpose, we match all defects into pairs of one *N* = 5 and *N* = 7 defect (Methods). We consider the nearest-neighbour matches as dislocations (otherwise, disclinations) and link their occurrences to trajectories using trackpy^46^. We provide a detailed visualization of the pair matching in Supplementary Fig. 3, including a more generalized defect clustering in good agreement with previous simulation results^47^. Figure 3c shows the histograms of the found dislocation displacements *Δ* within 62.5 ms for snapshots (i)–(iii) by using statistics of a total of 31 s around each snapshot. We find that in the solid regime (snapshot (i)), the defects are stable and may only rarely hop by a nearest-neighbour connection *r*_0_. In the hexatic regime (snapshot (ii)), nearest-neighbour hopping becomes more frequent and displacements different from *r*_0_ occur. The inset of snapshot (ii) (Fig. 3a) may serve as an illustration. The splitting of one dislocation into two causes a displacement of *r*_0_/2 due to the associated reorientation, whereas dislocation pairs changing between two horizontal and two vertical pairs contribute with displacements of $\sqrt{{r}_{0}}/2$ each. In the liquid regime (snapshot (iii)), an almost continuous distribution of displacements for the identified dislocations arises due to the complex rearrangements and interactions of defect clusters, signalling the unbinding of topological defects.

**Fig. 4:**
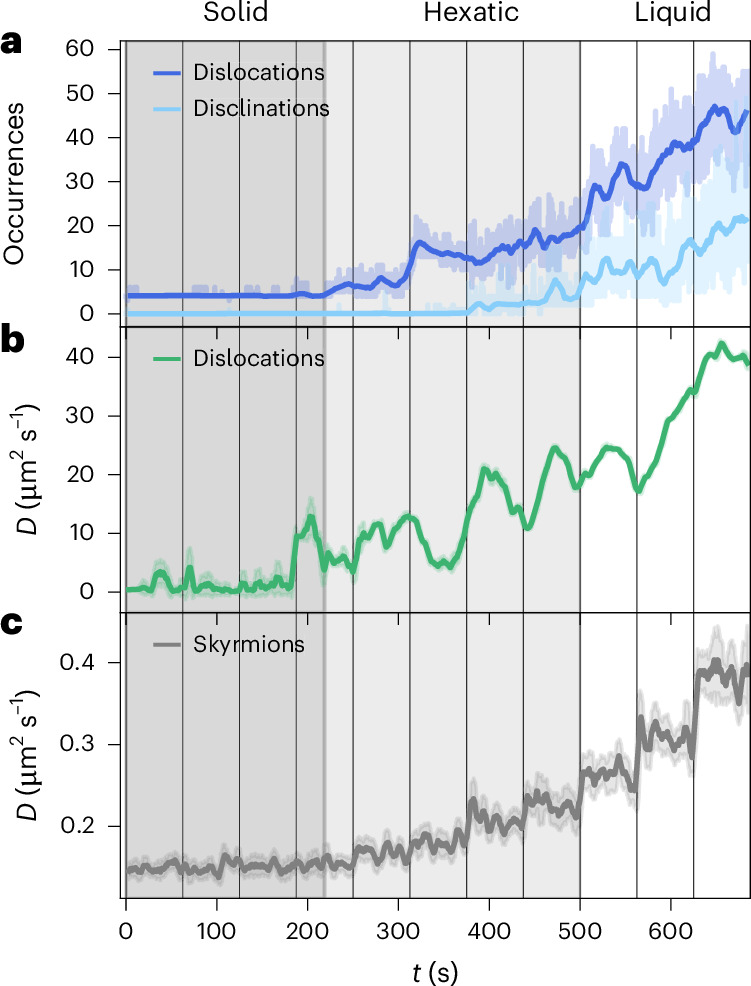
Time-resolved defect dynamics. **a**, Number of dislocations and disclinations observed during the melting for each frame (shaded) and a rolling mean over 6.25 s (100 frames). **b**,**c**, The diffusion coefficient *D* of the dislocations (**b**) is up to two orders of magnitude larger than the one for skyrmions (**c**). The solid lines represent the mean of the diffusion constant fit for a sliding window of 6.25 s (100 frames); the shaded area denotes the corresponding standard deviation. The dark-grey, light-grey and white backgrounds represent the predetermined solid, hexatic and liquid regimes, respectively. Source data

Our full real-time and real-space analysis allows us to ascertain the quantitative evolution of dislocations and disclinations (Fig. 4a). We find that the occurrence of dislocations (beyond the ones existing initially due to incommensurability and a vacancy) starts as predicted by theory at the transition from solid to hexatic. The occurrence of disclinations starts at the transition from the hexatic to liquid regime, also in good agreement with the KTHNY theory.

Finally, the identification of dislocations as topological quasi-particles throughout the melting process allows us to investigate their dynamics. From the mean squared displacement (MSD) of the dislocations (Supplementary Video 4), we fit the diffusion coefficient *D* (Methods), as shown in Fig. 4b. We find that for the dislocations, *D* is almost constant in the solid regime and increases drastically when transitioning from solid to hexatic due to dislocation unbinding. Within the hexatic regime, *D* continues to increase slightly as fluctuations increase. When entering the liquid regime, *D* starts increasing drastically again due to complex defect rearrangements. For comparison, we also determine the diffusion coefficient of skyrmions (Fig. 4c). For skyrmions, *D* increases continuously during melting; however, it does not change its behaviour qualitatively at the found transitions, highlighting the key role of topological defects in mediating melting. As the underlying mechanism, we see that the increasing skyrmion diffusivity due to a reduced packing fraction enables the formation and rearrangement of topological defects, which drastically speeds up the dislocation dynamics. During melting, *D* for the dislocations becomes up to two orders of magnitude larger than for the skyrmions forming the underlying lattice. This can be explained by the fact that dislocations are second-order (meta-) quasi-particles of the lattice (comprising quasi-particle skyrmions) and can, thus, hop significantly even if skyrmions are only slightly displaced. The fact that the dynamics of topological point defects can exceed the particle dynamics has been previously demonstrated for vacancies and interstitials^48,49^. Such point defects do not break QLRO and can also occur in our skyrmion lattice. An example of a quadruple vacancy is discussed in Supplementary Note 3. For 2D melting, however, the additional emergence of topological defects and especially the dynamics due to their dissociation is the key ingredient.

**Fig. 5:**
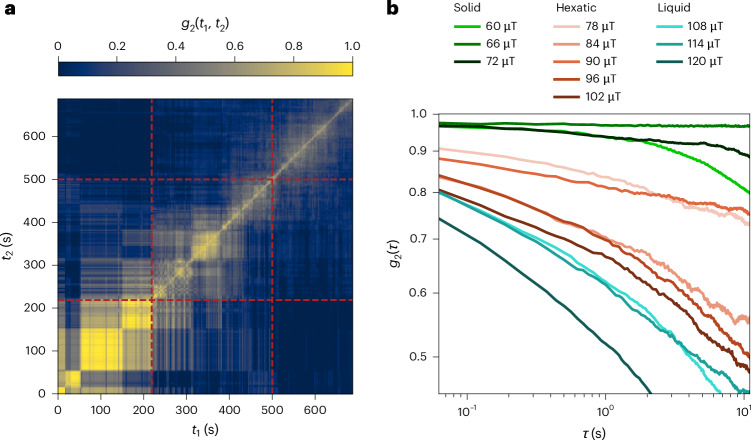
Time correlation of defect occurrences. **a**, Two-time correlation map of the Pearson correlation coefficient *g*_2_(*t*_1_, *t*_2_) correlating the defect occurrences of times *t*_1_ and *t*_2_ per skyrmion. The red dashed lines indicate the previously determined transitions from solid to hexatic and hexatic to liquid. The correlation generally decreases over time, with steps at the transitions. **b**, One-time correlation function *g*_2_(*τ*) as a function of delay *τ* = *t*_2_ – *t*_1_ for every magnetic-field interval of 62.5-s (1,000-frame) length. For increasing field throughout melting, *g*_2_(*τ*) decays faster, corresponding to an enhanced dynamics of topological defects. Source data

Although dislocation identification and tracking work generally well until deep in the hexatic regime, they become increasingly unstable at the onset of the liquid regime (Methods). Therefore, we also provide a different approach to access the defect dynamics by assessing the time correlation *g*_2_ of defect occurrences per skyrmion at times *t*_1_ and *t*_2_ (refs. ^50–52^) in Fig. 5 (Methods). As *g*_2_ decays increasingly fast throughout melting, we corroborate that the dynamics of defects keeps increasing—also throughout the liquid regime.

## Conclusions

In conclusion, we find the on-the-fly tunability of our system to be key to provide two unique and independent methods to controllably drive a 2D skyrmion lattice to disorder via a hexatic regime. We exploit a developed geometric confinement to locally stabilize the lattice, allowing us to capture the full intrinsic dynamics. Due to the rich dynamics and flexibility, real-time and real-space Kerr microscopy yields the necessary insights to understand the 2D melting process. We capture the emergence and dynamics of the characteristic topological defects mediating the phase transitions in the KTHNY theory and quantify the dynamics of the involved dislocations. Therefore, we image the so far unrevealed key mechanism of 2D melting, including a meta-quasi-particle diffusion coefficient of the dislocations that is found to be orders of magnitude higher than skyrmion diffusion. We quantify the correlation functions and shear modulus, which behave in excellent agreement with the KTHNY theory. Thus, our work provides a new approach to access the key dynamics in 2D melting and opens up new possibilities to study the intrinsic phase behaviour, boundary effects and identify the role of topological defects in 2D systems. In the future, the development of material stacks with further reduced pinning effects can even allow the use of skyrmion lattices for experimentally exploring 2D melting in the presence of a significant Magnus force^53^ as well as for determining the interaction potential of topological defects.

## Methods

### Magnetic multilayer stack

The Ta(5 nm)/Co_20_Fe_60_B_20_(0.9 nm)/Ta(0.07 nm)/MgO(2 nm)/Ta(5 nm) multilayer stack (layer thickness with an accuracy better than 0.01 nm) is deposited by d.c./radio-frequency magnetron sputtering using a Singulus Rotaris machine with a base pressure of 3 × 10^−8^ mbar. The hexagonal geometric confinement is patterned by electron-beam lithography followed by argon-ion etching.

Interfacial Dzyaloshinskii–Moriya interaction^54,55^ is mainly induced at the Ta/Co_20_Fe_60_B_20_ interface; the Co_20_Fe_60_B_20_/MgO interface causes perpendicular magnetic anisotropy. The Ta(0.08) dusting layer is used to not only balance the perpendicular magnetic anisotropy and Dzyaloshinskii–Moriya interaction^7,56^ to host skyrmions but also to optimize the energy landscape for skyrmion lattice formation and dynamics. We provide the OOP hysteresis loop in Supplementary Note 4 with Supplementary Fig. 4. The non-trivial topology of the observed bubbles is experimentally confirmed by spin–orbit-torque-driven skyrmion motion and supported by micromagnetic simulations^7,31,33^.

Furthermore, the skyrmion interaction potential has been demonstrated to be purely repulsive in the studied material stack^29^; in particular, we note that it is of a form in which the KTHNY transitions are predicted to occur^28^. In contrast, other materials can also lead to attractive skyrmion interaction potentials^57–59^. Although fundamentally, the existence of a Magnus force is a further unique property of skyrmions, the relative strength of the effect is, however, small in our system. It is roughly proportional to the ratio of the domain-wall width (10–20 nm) to the skyrmion core diameter (few micrometres)^33,60^ and, therefore, negligible in our system, resulting in a maximum skyrmion Hall angle of a few degrees^61^. Additionally, the hopping-like skyrmion dynamics in the non-flat-energy landscape is dominated by pinning forces^7,33,34^, suppressing the Magnus effect. Small skyrmions or (close-to) pinning-free diffusion systems can, however, lead to a sizable Magnus force, which is of special interest as topological defect dynamics in systems with a Magnus force and odd elasticity^53^ is an open question.

### Skyrmion stabilization and imaging

A commercially available evico magnetics Kerr microscope is used to establish magnetic contrast with a resolution of 300 nm in space and 62.5 ms in time, using a blue light-emitting diode light source and a charge-coupled device camera with a field of view of 200 × 150 µm^2^. Magnetic fields can be applied in both in-plane (IP) and OOP directions. The alignment of coils is optimized by aligning the shift of OOP hysteresis loops with and without an IP field. The OOP magnet is custom made to allow field control with sub-microtesla precision. The sample itself is placed onto a Peltier element directly on top of the coil for temperature control. The temperature is kept constant at 333.5 K and monitored by a Pt100 sensor directly next to the sample to ensure temperature stability better than 0.1 K.

Skyrmions are nucleated by applying an IP-field pulse, which saturates the sample in the IP direction at a constant OOP field. The resulting skyrmion lattice is equilibrated by an oscillating OOP magnetic field at 100 Hz with amplitudes up to 60 µT in addition to the constant OOP-field offset before measuring the obtained configuration.

We have direct and precise control over the skyrmion size via the applied OOP magnetic field^33,39,40^. The sizes of the individual skyrmions are detected by a machine learning-based pixel-wise classification^62^. Additionally, we can continuously tune the skyrmion diffusivity by sinusoidal OOP-field oscillation in addition to the offset field^9,34^. In the melting procedure presented in Figs. 1–4, we increase the external OOP-field offset every 62.5 s (corresponding to 1,000 frames) in steps of 6 µT. During each interval of 62.5 s, the field is kept constant to obtain reasonable statistics for every field value. In a constant external field, the skyrmion ensemble is in equilibrium. In the picosecond–nanosecond timescale of the magnetization dynamics, the intrinsic precessional dynamics of magnetization is always damped out on the timescales we investigate, whereas the thermally activated diffusive skyrmion dynamics takes place on the millisecond–second timescale that we investigate. The skyrmions also react fast (≲milliseconds)^34^ to field changes and the steps of 6 µT are very small—the system ordering responds to size changes typically faster than 1 s, apart from fluctuations close to the observed transitions. Therefore, we can treat the whole melting process to be in quasi-equilibrium. Accordingly, we have chosen the time intervals and field steps to preserve quasi-equilibrium and ensure stable measurement conditions during the whole melting protocol. However, oscillating fields—which are only used to initialize the skyrmion lattice order here, but which can also be used to destabilize the lattice order (Supplementary Fig. 4)—are expected to introduce non-equilibrium properties as they permanently drive skyrmion size changes.

### Quantification of 2D order

The translational order is quantified by the translational correlation function

1 $$\begin{array}{c}{G}_{{\rm{T}}}\left(r=\left|{{\bf{r}}}_{{{j}}}-{{\bf{r}}}_{{{k}}}\right|\right)=\left\langle {{\rm{e}}}^{-{\rm{i}}{\bf{G}}\cdot \left({{\bf{r}}}_{{{j}}}-{{\bf{r}}}_{{{k}}}\right)}\right\rangle \end{array},$$

averaging the link between two particle positions **r**_*j*_ and **r**_*k*_ with respect to a reciprocal lattice vector **G** over the distance *r*. The orientational correlation function

2 $$\begin{array}{c}{G}_{6}\left(r=\left|{{\bf{r}}}_{{{j}}}-{{\bf{r}}}_{{{k}}}\right|\right)=\left\langle {\psi }_{6}^{* }\left({{\bf{r}}}_{{{j}}}\right){\psi }_{6}\left({{\bf{r}}}_{{{k}}}\right)\right\rangle \end{array}$$

quantifies the orientational order based on the local orientational order parameter

3 $${\psi }_{6}\left({{\bf{r}}}_{{{j}}}\right)=\frac{1}{N}\mathop{\sum }\limits_{k=1}^{N}{{\rm{e}}}^{-{\rm{i}}6{\theta }_{{{j}}k}}$$

of a particle at position **r**_*j*_ with *N* nearest neighbours labelled *k* = 1 to *N*. *θ*_*jk*_ denotes the angle of the connecting vector **r**_*k*_–**r**_*j*_ with respect to an arbitrary axis^19^.

In a 2D solid, *G*_T_(*r*) decays algebraically as $\propto {r}^{-{\eta }_{{\rm{T}}}}$, signalling QLRO. When the exponent *η*_T_ reaches its critical value of 1/3, an exponential decay ∝exp(–*r*/*ξ*_T_) with correlation length *ξ*_T_ sets in; translational QLRO has disappeared^19^. In contrast, *G*_6_(*r*) is constant in a solid, but shows an algebraic decay $\propto {r}^{-{\eta }_{6}}$ when the translational order vanishes if orientational QLRO persists. Hence, orientational order is still present in what is referred to as the hexatic phase, which is unique to 2D systems^19,20^. When *η*_6_ reaches its critical value of 1/4, *G*_6_(*r*) becomes exponential (∝exp(–*r*/*ξ*_6_)) with correlation length *ξ*_6_, resulting in an isotropic liquid. At the transition from exponential to algebraic decay, the respective correlation lengths of both correlation functions diverge, causing the exponential term to vanish in the critical (QLRO) phases^19^.

Similar to the correlation functions in space, we calculate the orientational time correlation as

4 $$\begin{array}{c}{G}_{6}\left(\tau \right)=\left\langle {\psi }_{6}^{* }\left(t\right) {\psi }_{6}\left(t+\tau \right)\right\rangle \end{array}$$

as a function of time delay *τ*, which reveals the dynamics for every field interval^23^. The angle brackets represent the average over all particles and all starting times *t* within the interval of constant field. Theory suggests a constant behaviour of *G*_6_(*τ*) in the solid phase, algebraic decay in the hexatic phase and exponential decay in the liquid phase^23^. The hexatic and liquid phases are separated by a critical exponent *η*_τ_ = 1/8. Our results shown in Extended Data Fig. 1 match the theory qualitatively well. The predicted critical value of *η*_τ_ = 1/8 for an infinite system is, however, too large to match our scenario. We can attribute the enhanced time correlation in our experiment to the effects of confinement and non-flat-energy landscape.

Any lattice site with the number of nearest neighbours different from *N* = 6 is a topological defect. A dislocation is a pair of defects with opposite topological charge: one *N* = 5 and one *N* = 7 defect. In a solid, only a few dislocation pairs occur, which are tightly bound and of opposite orientation. The orientation of a dislocation is specified by the Burgers vector. The Burgers vector is determined as the missing vector when encircling a dislocation counterclockwise with a set of lattice vectors, which would yield a closed path in a perfect lattice. At the transition point separating the solid from the hexatic phase, the dislocation pairs unbind and proliferate. This formation of isolated free dislocations causing the loss of translational QLRO is measurable macroscopically as a vanishing shear modulus *µ*. At the transition point separating the hexatic from the liquid phase, the dislocations eventually unbind and proliferate into two isolated disclinations^18,19^.

### Data analysis

For the detection of skyrmions from the greyscale video and linking them to trajectories, we use the trackpy package^46^ in Python. The obtained positions are used for every skyrmion to determine the local order parameter *ψ*_6_ and its nearest neighbours applying a Voronoi tessellation, which automatically determines the lattice defects. Skyrmions at the edge of the system are neglected for the analysis of *ψ*_6_ and lattice defects as their position at the edge produces artefacts in the Voronoi tessellation^63^.

For all skyrmions that are not located at the edge of the system, we determine a value for *G*_T_ and *G*_6_ with respect to all other skyrmions. We bin the values of the respective correlation and perform an average in every bin, resulting in the distance-dependent correlation functions *G*_T_(*r*) and *G*_6_(*r*) (Fig. 1d–i). The determination of the correlation function works for single-frame images; however, we average the correlation functions of ten consecutive frames (over 0.625 s) to reduce noise significantly. Therefore, all the plots and fits of the correlation functions are performed on the averaged data. To determine the decay of the translational correlation function, we fit *G*_T_(*r*) with a power-law decay $\propto {\left(r{/r}_{0}\right)}^{-{\eta }_{{\rm{T}}}}$ as a function of distance *r* in units of the skyrmion lattice constant *r*_0_. We use the initial power-law fit to determine if the system is translationally ordered (*η*_T_ below a critical value of 1/3) or not (*η*_T_ > 1/3). In disorder, however, the exponent *η*_T_ is no longer well defined since the decay of *G*_T_ is now solely exponential. Therefore, for the disordered cases, we fit the exponential (∝exp(–*r*/*ξ*_T_)) instead of the power law. Since the exponential term is technically also present in the ordered critical regime, we also fit an exponential for the occurrences of *η*_T_ < 1/3, but as an additional factor to the power law. We use this additional factor in the fit as confirmation that the correlation length *ξ*_T_ becomes infinite in the ordered regime. For the orientational correlation function *G*_6_, we proceed analogously to determine the exponent *η*_6_ as well as the correlation length *ξ*_6_. However, the orientational correlation has a different critical value of *η*_6_ = 1/4, which we use to determine whether the system is orientationally ordered (*η*_6_ < 1/4) or not (*η*_6_ = 1/4) and whether we fit the exponential as an additional factor to or instead of the power law, respectively.

In our system, we lack the possibility to apply stress forces to directly measure the elastic moduli. Instead, we analyse the local deformations of the lattice in real space to estimate the shear modulus *μ* (refs. ^41,42,64^). As the reference lattice, we use a central skyrmion with six perfectly arranged nearest neighbours at positions ${{\bf{X}}}_{{\rm{i}}}^{{\rm{ref}}}$ with average lattice spacing. To this reference, we fit a local deformation tensor ${{{\delta }}}$ for every skyrmion and its neighbours in the experimental lattice, such that the squared distance

5 $${d}^{\;2}=\sum _{i}|\left({\delta } {{\bf{X}}}_{{\rm{i}}}^{{\rm{ref}}}\right)-{{\bf{X}}}_{{\rm{i}}}^{\exp }{|}^{2}$$

between experimental lattice positions ${{\bf{X}}}_{{\rm{i}}}^{\exp }$ and the tweaked reference is minimized. To extract the shear component, we decompose ${\delta }={\epsilon}+{{{R}_{\alpha }}}$ to a symmetric strain tensor ${\epsilon }$ and an anti-symmetric rotation ${{{R}_{\alpha }}}$ by an angle *α*. The diagonal elements of ${{\epsilon }}$ describe the strain along *x* and *y*, whereas the off-diagonal element is the shear component. In case of linear elasticity, a shear deformation is associated with a shear energy ${E}_{{\rm{shear}}}=\frac{1}{2}{(2{\epsilon }_{{xy}})}^{2}V\cdot \mu$, where *V* denotes the volume over which the shearing takes place (area spanned by the nearest neighbours in our case). Assuming a Boltzmann distribution *P*(*E*) ∝ exp(–*E*/*k*_B_*T*) of the shear energy at temperature *T*, we fit *µ* as the slope of

6 $$\begin{array}{c}\log \left[P\left(E\;\right)\right]=-\frac{{E}_{{\rm{shear}}}}{{k}_{{\rm{B}}}T}+{{\rm{constant}}}=\mu \left[\frac{1}{2}{\left(2{\epsilon }_{{x\;y}}\right)}^{2}\frac{V}{{k}_{{\rm{B}}}T}\right]+{{\rm{constant}}}\end{array}$$

when calculating a histogram over the square bracket as a measure of the logarithm of the shear energy distribution. The procedure requires the assumption of linear elasticity, which becomes less applicable in a less dense system, especially in liquid. Therefore, the shear modulus does not vanish completely during melting. Also, the distribution of shear energies associated with the determined deformations is not perfectly Boltzmann like, as already observed for colloid systems^42^. Since the dependence is not perfectly linear, we perform a set of fits over different ranges and use the standard deviation as error of the mean value.

The determination of topological defects follows directly from the Voronoi tessellation used for calculating the local ordering. Every skyrmion with a number of nearest neighbours *N* different from 6, which is not located at the edge of the system, is identified as a lattice defect. Since defects in the solid and hexatic regimes almost only occur pairwise, identifying those pairs as dislocations is trivial. However, transitioning to a liquid, complex clusters of defects evolve. The complex appearance, including the interactions between defect clusters, makes the identification of the formal connection between defects impossible. To analyse the further evolution of defects, we establish a simplified approach of identifying pairs of defects. To every 5-defect *i*, we assign exactly one 7-defect *j* and take the distance between the defects as *d*_*ij*_. To establish unique pair connections, we minimize the total square distance

7 $$\begin{array}{c}{d}_{{\rm{tot}}}^{\;2}=\mathop{\sum }\limits_{{ij}}{d}_{{ij}}^{\;2}\end{array}$$

associated with all possible connections *ij* using the Hungarian method^65^. We identify a determined defect pair as a dislocation if the corresponding *d*_*ij*_ is a nearest-neighbour connection; otherwise, we identify the two connected defects as two disclinations. To study the dislocation dynamics, we keep only the centre of mass of all the identified dislocations and link them to trajectories with trackpy^46^. Note that the 5/7-defect pair matching as well as the linking of dislocation trajectories work generally well until deep in the hexatic regime as defects always occur in pairs and do not fully dissociate. At the onset of the liquid regime, however, disclinations and complex defect clusters start occurring and make the formally correct matching and evolution of defect pairs inaccessible. With our approach being purely based on distance minimization, we, therefore, expect a possible systematic error in the quantification of defect dynamics from the onset of the liquid regime, whereas the increased dynamics as a direct consequence of defect fluctuations, rearrangements and dissociation is still reflected.

To evaluate the diffusion coefficient of the skyrmions at different times of the measurement, we determine the MSD as

8 $$\begin{array}{c}{\rm{MSD}}\left(t\right)=\left\langle {\left[{\bf{r}}\left(t\right)-{\bf{r}}\left({t}_{0}\right)\right]}^{{\rm{2}}}\right\rangle =2{dDt}\end{array}$$

by calculating the square distance of skyrmion position **r** at time *t* relative to the position at the time of initial occurrence *t*_0_ and take the average over all skyrmions. The MSD is further related to the dimensionality *d* of the system (here *d* = 2) and *D* over *t* in the case of normal diffusion^7,31,34^. Since we want to determine *D* at any time *t*_0_ with reliable statistics, we consider all trajectories present in a 10-s time window around *t*_0_ and use the time of first occurrence as *t*_0_. We then fit the first 1 s of the resulting MSD to determine *D*. For the dislocations, we proceed analogously but use all the trajectories occurring in a time window of 31 s around *t*_0_ to fit *D* for statistical reasons because there are significantly fewer dislocations than skyrmions.

To correlate defect occurrences of time, for every skyrmion *n* at time *t*, we associate a variable

9 $$\begin{array}{c}{u}_{n}\left(t\right)=\left\{\begin{array}{cc}1 & \left({\rm{no\; defect}}\right)\\ 0 & \left({\rm{is\; defect}}\right)\end{array}\right.\end{array}$$

to be correlated. We calculate the Pearson correlation

10 $$\begin{array}{c}{g}_{2}\left({t}_{1},{t}_{2}\right)=\frac{{\left\langle \left[u\left({t}_{1}\right)-{\rm{\mu }}\left({t}_{1}\right)\right] \left[u\left({t}_{2}\right)-{\rm{\mu }}\left({t}_{2}\right)\right]\right\rangle }_{n}}{\sigma \left({t}_{1}\right) \sigma \left({t}_{2}\right)}=\frac{{\left\langle u\left({t}_{1}\right) u\left({t}_{2}\right)\right\rangle }_{n}-{\left\langle u\left({t}_{1}\right)\right\rangle }_{n}{\left\langle u\left({t}_{2}\right)\right\rangle }_{n}}{\sigma \left({t}_{1}\right) \sigma \left({t}_{2}\right)}\end{array}$$

for every pair of times *t*_1_ and *t*_2_ by averaging over all skyrmions *n* (refs. ^50–52^). Here *μ* and *σ* represent the mean and standard deviation of *u* at the respective time. The corresponding two-time correlation map is shown in Fig. 5a. The correlation decreases over time during the melting and one can observe more rapid changes in the time regions of the previously determined transitions (Fig. 5a, dashed red lines).

By averaging over equal time delays *τ* = *t*_2_ – *t*_1_, we convert the two-time correlation map to a one-time correlation function *g*_2_(*τ*) for every interval of constant magnetic field (as the field is changed stepwise every 62.5 s). Although *g*_2_(*τ*) (Fig. 5b) stays almost constant for the fields representing the solid regime, it decays notably and increasingly rapidly throughout the melting process. As the decay of *g*_2_(*τ*) is directly related to the dynamics of the underlying feature^51,52^—that is, the topological defects in this case—this corroborates that the defect dynamics keeps increasing throughout the melting procedure.

## Online content

Any methods, additional references, Nature Portfolio reporting summaries, source data, extended data, supplementary information, acknowledgements, peer review information; details of author contributions and competing interests; and statements of data and code availability are available at 10.1038/s41565-025-01977-2.

## Supplementary information

Supplementary Information Supplementary Notes 1–4 and Figs. 1–4.

Supplementary Video 1 Kerr video of skyrmion lattice melting by shrinking the skyrmions: Kerr microscopy video (real time, 16 fps) of a skyrmion lattice melting from solid to liquid via hexatic. To induce the melting, the applied magnetic field is increased every 62.5 s to shrink the skyrmions. The video is used for the analysis shown in Figs. 1–5. Edge length of the hexagonal pattern: 100 µm. The uncompressed full-resolution video is available in ref. ^38^.

Supplementary Video 2 Kerr video of skyrmion lattice melting by increasing diffusivity: Kerr microscopy video (real time, 16 fps) of a skyrmion lattice melting from hexatic to liquid. Magnetic-field oscillations of increasing amplitude (stepwise every 62.5 s) cause the skyrmion dynamics to increase and destabilize the lattice order. Therefore, the skyrmions are also destabilized and start annihilating. The video is used for the analysis shown in Extended Data Fig. 2. Edge length of the hexagonal pattern: 100 µm. The uncompressed full-resolution video is available in ref. ^38^.

Supplementary Video 3 Topological defect dynamics during the melting: animation of topological defects (real time, 16 fps) characterized by the number of lattice neighbours during the melting, as extracted from Supplementary Video 1.

Supplementary Video 4 Dislocation dynamics during the melting: animation of the dislocations (real time, 16 fps) in the melting extracted from Supplementary Video 3. The black line segments visualize the identified connections between matched *N* = 5 and *N* = 7 defects identified as dislocations. The purple lines show the respective trajectories of the dislocations over the past 1 s.

## Source data

Source Data Fig. 1 Statistical source data for Fig. 1.

Source Data Fig. 2 Statistical source data for Fig. 2.

Source Data Fig. 3 Statistical source data for Fig. 3.

Source Data Fig. 4 Statistical source data for Fig. 4.

Source Data Fig. 5 Statistical source data for Fig. 5.

Source Data Extended Data Fig. 1 Statistical source data for Extended Data Fig. 1.

Source Data Extended Data Fig. 2 Statistical source data for Extended Data Fig. 2.

Source Data Extended Data Fig. 3 Statistical source data for Extended Data Fig. 3.

## Data Availability

The data supporting the findings of this study are available within the article and Supplementary Information. These data are also available via Zenodo at 10.5281/zenodo.15472065 (ref. ^38^). Source data are provided with this paper.
