# Supplementary material for: Real-time observation of topological defect dynamics mediating two-dimensional skyrmion lattice melting
Source: Nat Nanotechnol. 2025 Aug 4;20(10):1405–11. doi: 10.1038/s41565-025-01977-2 (PMC12534186; doi:10.1038/s41565-025-01977-2)
Supplement: Supplementary file 1 — Supplementary Notes 1–4 and Figs. 1–4. [file 41565_2025_1977_MOESM1_ESM.pdf]

nature nanotechnology

Supplementary information https://doi.org/10.1038/s41565-025-01977-2

Real-time observation of topological defect dynamics mediating two-dimensional skyrmion lattice melting

In the format provided by the authors and unedited

Content

Supplementary Note 1: Finite Size Effects in the Experiment 2
Supplementary Fig. 1 3
Supplementary Note 2: Finite Size Effects in Simulations 4
Supplementary Fig. 2 6
Supplementary Note 3: Defect Clustering and Defect Pair Matching 7
Supplementary Fig. 3 8
Supplementary Note 4: Hysteresis Loop 9
Supplementary Fig. 4 9
References 9

Supplementary Note 1: Finite Size Effects in the Experiment

Since the experimentally observed lattice is a finite system, it is expected to exhibit finite size effects. In contrast, measures to classify phases in KTHNY are derived for infinite systems. Therefore, we investigate in the following how our results align with those measures.

Analyzing the behavior of G_T and G_6 at larger distances (Fig. S1a and b, respectively; both for snapshots I-III), we see that the correlation functions can increase with distance due to the lattice stabilization by the boundary, especially for distances beyond ten nearest neighbor distances r_0. In contrast to Fig. 1, we use a semilogarithmic plot to highlight the larger distances. The effect is stronger for the orientational correlation since the confinement with its commensurate shape provides primarily orientational stabilization. We observe the boundary effect on G_T starting at distances of approximately 12 r_0; for G_6, an influence is visible already at 7 r_0. We therefore use those distances as limits for the fit range when we determine the decay exponents η_T and η_6, respectively.

According to KTHNY theory, the correlation functions are expected to change their functional behavior qualitatively between power-law and exponential at the critical point determined by the critical exponents. We therefore compare the reduced χ^2 of pure power-law and pure exponential fits during the entire melting procedure. For each distance bin of the correlation functions, we use the mean and standard deviation from the performed average over 10 frames (0.625 s) for the fit. In Fig. S1c, we show that a power-law fit for G_T is indeed more favorable in the predetermined solid regime (dark-gray shading). Entering the hexatic regime (light-gray shading), the exponential becomes more favorable. Hence, the critical value of 1/3 does not appear to be affected by finite size effects. At t>450 s, the power-law fit yields decreasing values χ^2 and seems to become favorable again; however, this is only because of the small values of G_T deep in disorder, which therefore become increasingly prone to noise and the present boundary effects. For G_6., χ^2 of the power-law fit is always smaller than of the exponential, thus indicating better description by a power-law behavior for all times. However, due to the boundary effect seen in Fig. S1b, the fit region is strongly limited and yields maximum 7 points; especially, we lack values over larger distances, which are decisive to analyze the functional behavior. In contrast to the critical QLRO regime, we therefore do not find a clear preference for either a power-law or exponential behavior but our results remain inconclusive regarding the exact form. In the experiment presented in Fig. S3h however, we find that G_6 is indeed exponential as soon as the stabilization due to the boundary is overcome; there, with sufficient diffusion.

For the translational correlation, we furthermore notice an anisotropy of the crystal order. Fig. S1e shows the Fourier transform (FFT) of the Kerr microscopy snapshot I. We clearly see discrete peaks (even in higher order) in sixfold symmetry as expected for a hexagonal lattice. The first-order peaks determine the reciprocal hexagonal lattice vectors used for the calculation of G_T. The stripes through the center (in six-fold symmetry) are an artefact caused by the hexagonal shape of the confinement. As a feature of the finite nature of our system, G_T. can evolve differently depending on the choice of the lattice vector used. Fig. S1f provides a comparison for the usage of the three lattice vectors in G_T resulting in differently fast decays. The anisotropy I only possible on finite scales and depends on the specific location of lattice defects.

Supplementary Fig. 1

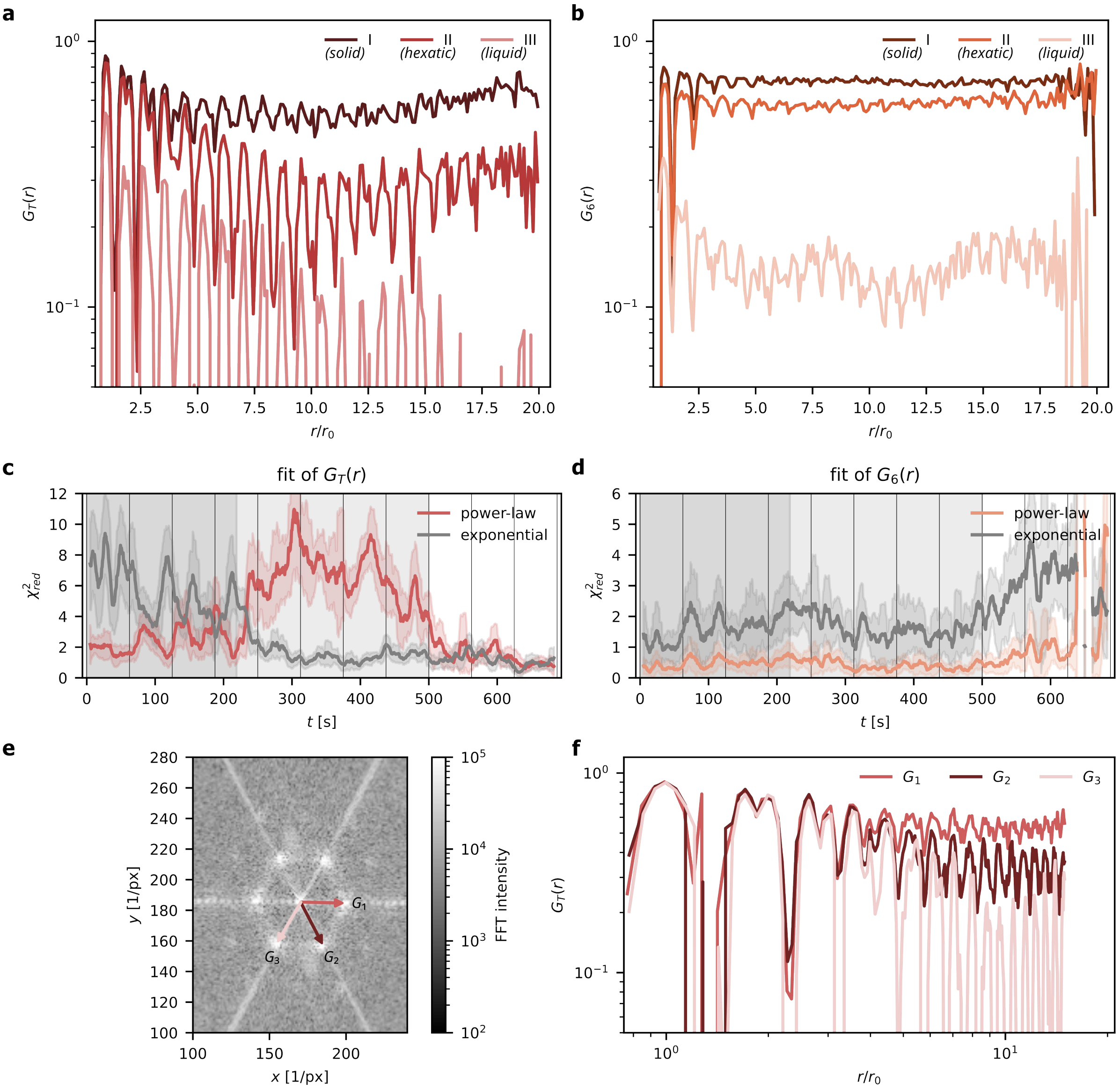

Supplementary Fig. 1. Analysis of Finite Size Effects in the Experiment. a-b G_T (a) and G_6 (b) for single-frame snapshots of snapshots I-III (as examples for solid, hexatic, liquid) as presented in Fig. 1, but up to the maximum system distance of ~$20r_0$. Note that in contrast to Fig. 1, we show a semilogarithmic plot to highlight the effect of larger distances: Due to the orientational stabilization by the hexagonal confinement, the correlation functions may increase again due to large distances being increasingly affected by boundary effects. **c-d** Reduced χ^2 for fitting a pure power-law (colored) or exponential (gray) to the correlation functions G_T (c) and G_6 (d) throughout the melting over time t. Average and standard deviation over 10 s are drawn. The dark-gray, light-gray and white shading represent the solid, hexatic and liquid phase, respectively. **e** Fourier transformed frame image of snapshot I exhibiting sixfold symmetry and determining the reciprocal lattice vectors G_1-G_3 (drawn as arrows). The six-fold lines are due to the hexagonal geometry of the system. **f** Due to the finite system size and the specific lattice defect configuration present, the translational correlation function may depend on the choice of lattice direction (G_1-G_3) as presented here for snapshot I.

Supplementary Note 2: Finite Size Effects in Simulations

To analyze finite size effects and their dependence on the system size, we perform computer simulations in the Thiele model[1] applied to magnetic skyrmions. In the case investigated in this article, the equation of motion reads[2,3]

$$-\gamma\mathbf{v} - G_{\mathrm{rel}}\gamma\mathbf{e}_z \times \mathbf{v} + \mathbf{F}_{\mathrm{therm}} + \mathbf{F}_{\mathrm{SkSk}}(\{\mathbf{r}\}) + \mathbf{F}_{\mathrm{SkBnd}}(\mathbf{r}) = 0 \qquad (S1)$$

where $\mathbf{r}$ is the skyrmion position, $\mathbf{v}$ the skyrmion velocity and $\{\mathbf{r}\}$ indicates the set of all skyrmion positions. γ indicates the damping (in the context of a Molecular Dynamics simulation, not to be confused with the Gilbert damping) and G_{rel} is the relative Magnus force amplitude which is calculated as tangent of the skyrmion Hall angle. In this article, G_{rel} and thereby the Magnus force is set to zero as the Magnus force affects only dynamics of the system but not static properties such as ordering. γ is set to 1 (in simulation units). $\mathbf{F}_{\mathrm{therm}}$ is thermal Gaussian white noise satisfying the fluctuation-dissipation theorem at a temperature of $k_{\mathrm{B}}T$=1 (in simulation units). $\mathbf{F}_{\mathrm{SkSk}}$ and $\mathbf{F}_{\mathrm{SkBnd}}$ indicate skyrmion-skyrmion and skyrmion-boundary repulsion. The skyrmion-skyrmion interaction is approximated by a $V(r)=r^{-8}$ potential with a cutoff distance of 1.8 simulation units[4] and the skyrmion-boundary interaction is using a fully repulsive Lennard-Jones potential

$$V_{\mathrm{LJ}}(r) = 4\varepsilon\left[\left(\frac{\sigma}{r}\right)^{12} - \left(\frac{\sigma}{r}\right)^{6} + \frac{1}{4}\right] \qquad (S2)$$

with $r_{\mathrm{cut}}=2^{1/6}$ and $\varepsilon=\sigma=1$. Skyrmion interaction potentials for similar materials have previously been extracted using Iterative Boltzmann Inversion (IBI)[2], where no assumptions on the potential form had been made. The distances and the exact potential form are not directly extracted from the experiment in this case as such high densities generally cause artefacts in the IBI[2]. Instead, the exponent n=8 is determined from a slightly less dense skyrmion liquid[5]. Comparing with other measurements in similar material stacks, we have made sure that the exponent changes by less than 1 for size variations two times larger than covered in this investigation – thus not affecting the theoretically predicted phase behavior[4]. In particular, the r^{-8} potential features well-known liquid-hexatic and hexatic-solid phase transitions in continuum[4]. To explain the lattice melting induced by shrinking the skyrmions (i.e., the packing fraction), we simulate the system at different skyrmion densities. Accordingly, we adjust the interaction radius of the skyrmions, but not the form of the potential given by the approximately constant exponent.

Densities ρ in this simplified model are defined as skyrmions per unit length squared in simulation units. The equations of motion were integrated using an Euler algorithm

$$\mathbf{r}(t + \Delta t) = \mathbf{r}(t) + \mathbf{v}(t)\Delta t \qquad (S3)$$

with a time step of $\Delta t=10^{-4}$ implemented in the *HOOMD-blue* software package[6]. The system is initialized with either perfect hexagonal order (for commensurate numbers) or hexagonal order with a few particles missing (for non-commensurate numbers) and equilibrated for 10^6 steps before running for 10^7 steps with the trajectory saved every 10^4 steps. Correlation functions are calculated and fitted individually for every saved step. Simulations of non-commensurate numbers are all averaged over at least 10 independent runs with independent initialization. Results of these simulations are presented as the mean over each individual simulation. Commensurate simulations use only one simulation run (except for 11 and 12 skyrmions per edge where we perform 10 runs) as there is only one specific starting condition and for large number a significantly longer computation time is required.

The main approximations of the Thiele model include that skyrmions are described as perfectly circular and of constant size as well as neglecting skyrmion creation and annihilation. These approximations are justified by the small amount of size polydispersity (standard deviation of the

diameter: 11 %) as well as deformations found in the experiments. Also, the system investigated does not feature spontaneous skyrmion creation; skyrmion annihilation is only observed deep in the liquid regime. This makes the Thiele model applicable for a description of the skyrmions used in this article.

In Fig. S2a, we show for computer simulations of a hexagonal system with commensurate numbers of skyrmions (centered hexagonal numbers) that the hexagonal boundary condition stabilizes QLRO in the system. The stabilization becomes stronger for smaller systems, causing QLRO to persist down to lower skyrmion density. We plot the fraction of states observed in the solid, hexatic and liquid regime, respectively. For smaller systems, transition regions between the different regimes at identical parameters also become larger. For larger systems, the influence of the finite size effects decreases, the regimes occur separated and the densities where the system transitions between the regimes approach the values for an infinite system[4].

In addition to the size scaling of the system, we also investigate how the commensurability of the particle number with respect to the confinement geometry[7] affects the ordering. Fig. S2b depicts similar simulations for various numbers of skyrmions around and between the commensurate (centered hexagonal) numbers of 397 (11 per edge) and 469 (12 per edge). As incommensurability enforces the existence of dislocations, it strongly suppresses translational QLRO, i.e. the solid regime; in return, the liquid and especially the hexatic regime are widened and enhanced due to quenched disorder[8,9]. Interestingly, we find a half-commensurate state alternatingly combining 11 and 12 skyrmions per edge, weakly stabilizing QLRO.

The experiments presented in the main text are performed with 401 skyrmions, i.e. slightly off commensurability. Indeed, the system exhibits the three different, well-separated regimes of order and is thus in good agreement with the simulations and KTHNY theory in general. In particular, we observe a relatively wide parameter space for the hexatic regime due to slight incommensurability and low pinning, while still benefitting from sufficient local stabilization due to the geometric confinement.

Supplementary Fig. 2

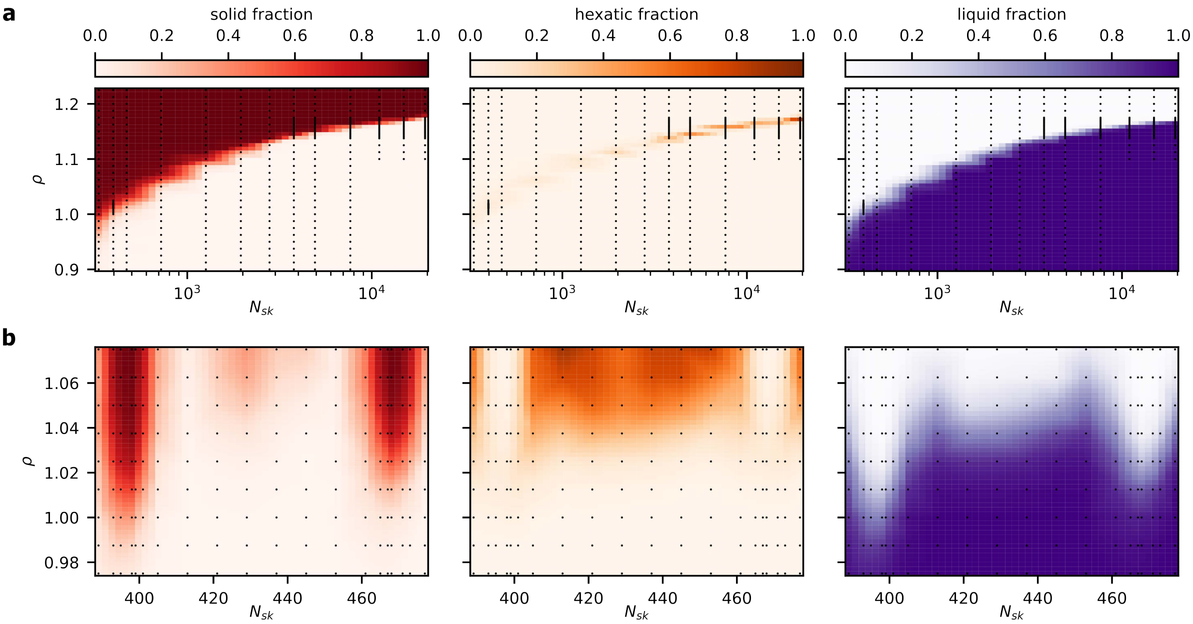

Supplementary Fig. 2. Ordering for different numbers of skyrmions in a hexagonal confinement. **a** Ordering regimes in computer simulations of skyrmions in the Thiele model for different particle densities ρ and system sizes using commensurate numbers of skyrmions N_{sk} (centered hexagonal numbers) between 331 (10 skyrmions per edge) and 19441 particles (80 per edge). The black dots represent the parameters at which simulations are performed. The coloring of the parameter space denotes which fractions of the recorded simulations is in the respective regime; for visualization purposes, we show the linear interpolation of the determined fraction over the entire parameter space. The three regimes (solid, hexatic, liquid) exist for all system sizes and QLRO is stabilized by the hexagonal confinement: The smaller the system, the lower the density at which QLRO can still be stabilized; transition regions between different regimes also become larger. The square artefact between simulation parameters are due to the linear interpolation displayed on a logarithmic scale. **b** Analogously, ordering regimes of non-commensurate numbers of skyrmions. Between the commensurate states containing 397 (11 per edge) and 469 skyrmions (12 per edge), translational QLRO and thus the solid regime is strongly suppressed. Instead, the liquid and especially the hexatic regime are enhanced. In between, we find a half-commensurate state (alternating combination of 11 and 12 skyrmions per edge), QLRO is weakly stabilized.

Supplementary Note 3: Defect Clustering and Defect Pair Matching

To detect and classify defect clusters, an approach based on the Voronoi tessellation of the system is used. For each particle with a neighbor number less (greater) than N=6, its neighbors are checked and neighboring particles with more (less) than N=6 neighbors are considered an associated pair. If no neighbor with an opposing neighbor number defect is found, it is classed as an isolated disclination. Otherwise, all particles within a graph of shared associations are considered part of the same cluster. Clusters consisting of exactly one 5- and one 7-defect are classed as isolated dislocations. Dislocation pairs are defined as consisting of exactly two 5- and two 7-defects where each defect is neighbor to both of the opposing defects. Chains are defined as any cluster consisting of at least 3 particles, which are connected linearly only.

Based on the Voronoi tessellation of the particle positions, particles that do not have exactly 6 neighbors are identified as defects and neighboring opposing defects are combined to clusters. This allows for the identification of different types of defect clusters to compare them to theoretical predictions[13–16]. While theory predicts only dislocation pairs in the solid phase, four stable 5-7-defect pairs (dislocations) are present. As the number of particles (401) is not commensurate with the confinement (closest match would be 397), one dislocation is needed to fit the additional particles. In Fig. S5a, we find that the number of skyrmions along every edge (black dots) is 12 except for the right edge, which contains 13 skyrmions, as indicated by the surrounding arrows labelled with the number of edge skyrmions. The 13th skyrmion causes an additional crystal line (green dots), which ends at one dislocation. Instead of only the four excess skyrmions however, this additional line features eight skyrmions. That is, the other four skyrmions have to be missing elsewhere. Thus, the other three, encircled dislocations comprise a quadruple vacancy and are topologically trivial: their Burger's vectors (red arrows) span all three lattice vectors exactly once and add up to zero. In the inset, we compare the observed skyrmion occurrences inside this vacancy (overall gray Delauney triangulation forming the nearest-neighbor net) to the ideal case in which the surrounding lattice would continue (indicated by black grid). When we match every occurring skyrmion to one ideal lattice site (purple links), indeed four sites (open green circles) stay vacant, confirming the quadruple vacancy. The combination of the observed dislocation (due to the incommensurability) together with the quadruple vacancy as stable initial state is a consequence of the non-flat energy landscape. Consequently, the top dislocation is bound to the right edge and can in the solid regime only move up or down with a fixed distance of eight skyrmions to the edge (see Supplementary Video 3), or later interact with other defects. In the hexatic phase, dislocation pairs as well as chains proliferate, but defects are still bound in opposing pairs. The total number of defects again rises in the liquid phase and dislocations disassociate leading to isolated defects.

Supplementary Fig. 3

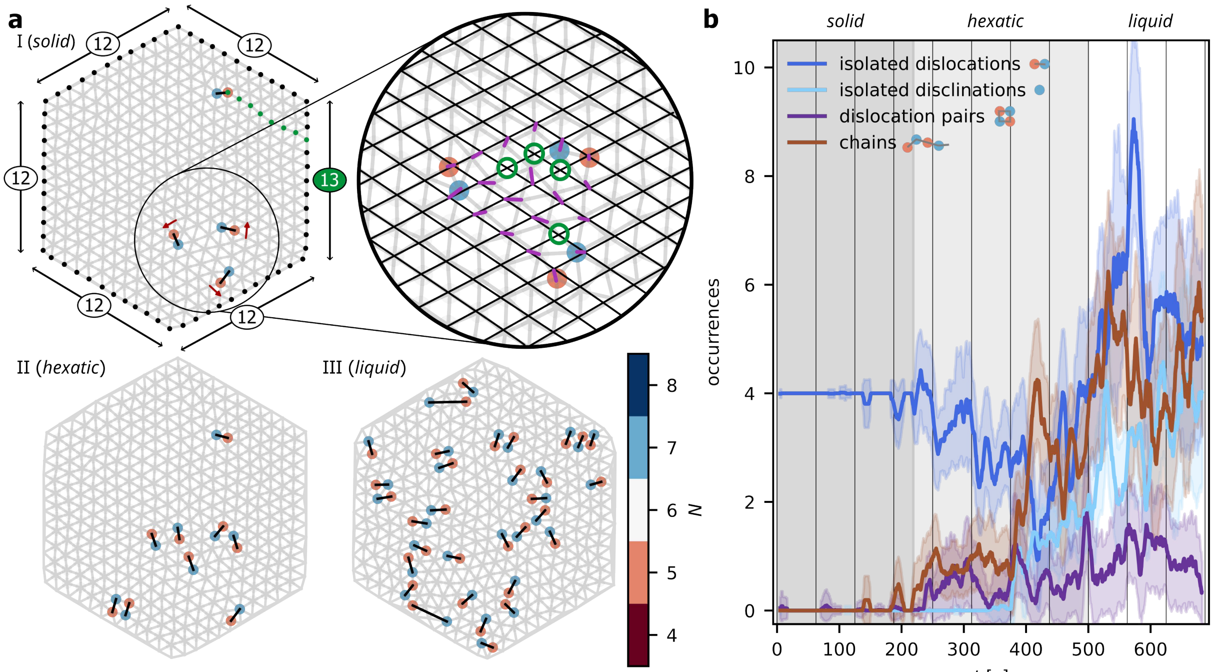

Supplementary Fig. 3. Defect Clustering and Pair Matching. a Defect pair matching for snapshots I-III. The black lines represent the connection within a pair. While the matching is trivial in the solid and hexatic regime, the matching in the liquid is not obvious and obtained by distance minimization. In snapshot I, we additionally note that every edge contains 12 skyrmions (black dots and number on arrows), except the right edge with 13 skyrmions. The 13th skyrmion causes the existence of an additional skyrmion row (marked in green), ending at a dislocation. The remaining three encircled dislocations are topologically trivial as their Burger's vectors (red arrows) cancel. They comprise a quadruple vacancy as the lattice has four excess skyrmions (401 instead of 397), but eight are bound to the top dislocation (green dots). The circle inset visualizes the appearance of the fourfold vacancy: if we at the vacancy map in purple every skyrmion to the ideal surrounding lattice (black lines), four sites remain unoccupied (green open circles). **b** Occurrences of more specific clusters of defects occurring during the melting (rolling average and standard deviation over 10 s).

Supplementary Note 4: Hysteresis Loop

We measure the OOP hysteresis loop in terms of the relative OOP magnetization m_z by the total intensity in Kerr microscopy over an OOP field (B) cycle. Note that the saturation field of the loop is below 200 µT, which is of the order of the earth magnetic field. We correct the surrounding magnetic offset field by shifting the hysteresis to be centered around zero. All field values given in the manuscript are corrected for the background field.

Supplementary Fig. 4

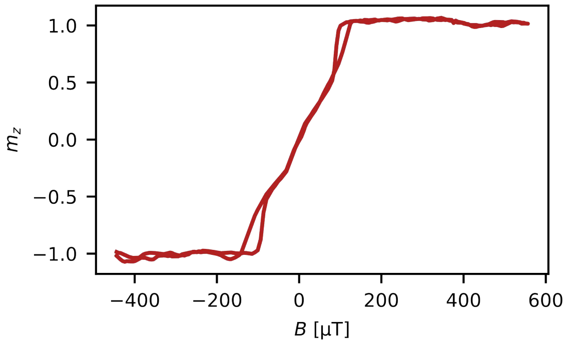

Supplementary Fig. 4. Hysteresis Loop. The red line shows the MOKE intensity corresponding to the relative magnetization m_z for a cycle of the OOP magnetic field B.

References

1. Thiele, A. A. Steady-State Motion of Magnetic Domains. *Phys. Rev. Lett.* **30**, 230–233 (1972).
2. Ge, Y. *et al.* Constructing coarse-grained skyrmion potentials from experimental data with Iterative Boltzmann Inversion. *Commun. Phys.* **6**, 1–6 (2023).
3. Brems, M. A. *et al.* Realizing Quantitative Quasiparticle Modeling of Skyrmion Dynamics in Arbitrary Potentials. *Phys. Rev. Lett.* **134**, 046701 (2025).
4. Kapfer, S. C. & Krauth, W. Two-Dimensional Melting: From Liquid-Hexatic Coexistence to Continuous Transitions. *Phys. Rev. Lett.* **114**, 035702 (2015).
5. Rothörl, J. Particle-based computer simulations of magnetic skyrmions. (Johannes Gutenberg-Universität Mainz, Mainz, 2024).
6. Anderson, J. A., Glaser, J. & Glotzer, S. C. HOOMD-blue: A Python package for high-performance molecular dynamics and hard particle Monte Carlo simulations. *Comput. Mater. Sci.* **173**, 109363 (2020).

7. Song, C. *et al.* Commensurability between Element Symmetry and the Number of Skyrmions Governing Skyrmion Diffusion in Confined Geometries. *Adv. Func. Mater.* **31**, 2010739 (2021).
8. Deutschländer, S., Horn, T., Löwen, H., Maret, G. & Keim, P. Two-Dimensional Melting under Quenched Disorder. *Phys. Rev. Lett.* **111**, 098301 (2013).
9. Nelson, D. R. Reentrant melting in solid films with quenched random impurities. *Phys. Rev. B* **27**, 2902–2914 (1983).
10. Zahn, K. & Maret, G. Dynamic Criteria for Melting in Two Dimensions. *Phys. Rev. Lett.* **85**, 3656–3659 (2000).
11. Gruber, R. *et al.* 300-Times-Increased Diffusive Skyrmion Dynamics and Effective Pinning Reduction by Periodic Field Excitation. *Adv. Mater.* **35**, 2208922 (2023).
12. Huang, P. *et al.* Melting of a skyrmion lattice to a skyrmion liquid via a hexatic phase. *Nat. Nanotechnol.* **15**, 761–767 (2020).
13. Kosterlitz, J. M. & Thouless, D. J. Long range order and metastability in two dimensional solids and superfluids. (Application of dislocation theory). *J. Phys. C: Solid State Phys.* **5**, L124 (1972).
14. Kosterlitz, J. M. & Thouless, D. J. Ordering, metastability and phase transitions in two-dimensional systems. *J. Phys. C: Solid State Phys.* **6**, 1181–1203 (1973).
15. Halperin, B. I. & Nelson, D. R. Theory of Two-Dimensional Melting. *Phys. Rev. Lett.* **41**, 121–124 (1978).
16. Nelson, D. R. & Halperin, B. I. Dislocation-mediated melting in two dimensions. *Phys. Rev. B* **19**, 2457–2484 (1979).
17. Lal, J., Abernathy, D., Auvray, L., Diat, O. & Grübel, G. Dynamics and correlations in magnetic colloidal systems studied by X-ray photon correlation spectroscopy. *Eur. Phys. J. E* **4**, 263–271 (2001).
18. Lhermitte, J. R. M., Rogers, M. C., Manet, S. & Sutton, M. Velocity measurement by coherent x-ray heterodyning. *Review of Scientific Instruments* **88**, 015112 (2017).

19. Klose, C. *et al.* Photon correlation spectroscopy with heterodyne mixing based on soft x-ray magnetic circular dichroism. *Phys. Rev. B* **105**, 214425 (2022).
20. Russell, E. R., Spaepen, F. & Weitz, D. A. Anisotropic elasticity of experimental colloidal Wigner crystals. *Phys. Rev. E* **91**, 032310 (2015).
